# Serum Interleukin (IL)-6, Lipid Profile, and Association With Disease Severity in Patients With Psoriasis: A Cross-Sectional Study

**DOI:** 10.7759/cureus.69599

**Published:** 2024-09-17

**Authors:** Soumya Ranjan Sahoo, Kuldip Das, Bharti Panda, Mamata Pandey, Madhusmita Acharya, Purnima Meher, Sumitra Bhoi

**Affiliations:** 1 Department of Biochemistry, Veer Surendra Sai Institute of Medical Sciences and Research, Burla, IND; 2 Department of Dermatology, Veer Surendra Sai Institute of Medical Sciences and Research, Burla, IND; 3 Department of Community Medicine, Veer Surendra Sai Institute of Medical Sciences and Research, Burla, IND; 4 Department of Multidisciplinary Unit, Veer Surendra Sai Institute of Medical Sciences and Research, Burla, IND; 5 Department of Physiology, Veer Surendra Sai Institute of Medical Sciences and Research, Burla, IND

**Keywords:** dyslipidemia, il-6, lipid profile, psoriasis, skin diseases

## Abstract

Objectives: This study estimated the lipid profile and inflammatory biomarker (interleukin-6, IL-6) levels in patients with psoriasis and explored the association between lipid profiles and inflammatory biomarkers (IL-6) in assessing the severity of the disease.

Methodology: This cross-sectional study was conducted in the Department of Biochemistry, in association with the Department of Dermatology, at the Veer Surendra Sai Institute of Medical Sciences and Research, Burla, from November 2022 to June 2023. A fully automated analyzer (Cobas C311, Roche Diagnostics, Mannheim, Germany) was used to estimate the lipid profile, while the enzyme-linked immunosorbent assay (Erba, Transasia Bio-medicals Ltd., Mumbai, India) was used to estimate the inflammatory marker (IL-6). Data were analyzed using Statistical Package for the Social Sciences version 21.0 (IBM Corp., Armonk, NY). An unpaired t-test was conducted to examine the relationship between two variables. A p value of <0.05 was considered significant. The correlation among IL-6, lipid profile, and psoriasis area severity index (PASI) score was analyzed using Pearson’s correlation coefficient. Quantitative data were expressed as means and standard deviations.

Results: Of the 102 study participants, 68 had psoriasis alone, and 34 had psoriasis with arthritis (psoriatic arthritis, PsA). Higher mean serum concentrations of IL-6, total cholesterol (TC), low-density lipoprotein, and triglycerides were found in patients with PsA compared to those with psoriasis alone. In patients with PsA, TC and IL-6 showed a strong positive correlation with the PASI score. In contrast, high-density lipoprotein showed a negative correlation with PASI (r = -0.036, p < 0.05).

Conclusion: In conclusion, our study revealed lipid profile abnormalities and increased IL-6 activity in PsA patients. By measuring inflammatory markers (IL-6) and lipid profiles early in the disease process, we can employ preventive strategies to better manage and improve survival and quality of life in patients with psoriasis.

## Introduction

Psoriasis is a chronic inflammatory dermatological disorder characterized by sharply demarcated, reddish, and scaly plaques, which mostly appear on the extensor prominences and scalp [1]. Psoriatic arthritis (PsA) is a complex chronic inflammatory disease usually associated with various clinical presentations, such as arthritis, enteritis, and dactylitis [2]. The prevalence of PsA among psoriasis patients varies from 6.25% to 48% in Western countries [3]. Psoriatic lesions are present in 60%-70% of patients, while in 15-20% of cases, arthritis precedes the onset of these lesions [4]. About 30% of PsA cases involve the fingers, toe joints, and tendons [5]. Moreover, the chronic inflammatory process in psoriasis may lead to metabolic disorders like dyslipidemia [6].

Interleukin-6 (IL-6) is a proinflammatory cytokine that activates the growth of dermal and epidermal cells in our body [7]. Several studies have concluded that serum IL-6 concentration is an essential parameter for treatment response and inflammation [8-10]. In people with psoriasis, serum IL-6 levels may be associated with skin and joint disease severity, as IL-6 triggers synovial inflammation and hyperplasia [11]. Continuous exposure to UV radiation and environmental stress factors can target our skin for oxidative injury, producing reactive oxygen species [12]. Long-term inflammation of psoriatic lesions releases various proinflammatory cytokines, including IL-6. This inflammatory process raises the levels of triglycerides (TG), total cholesterol (TC), low-density lipoprotein cholesterol (LDL-C), and very LDL-C in the blood. In addition to oxidative stress, atherosclerosis, dyslipidemia, and insulin resistance are common features of psoriasis [13]. The atherogenic profiles of individuals with psoriasis and PsA were examined, which find changes in lipid profile parameters, such as higher levels of TC, LDL-C, and TG in the blood.

Moreover, patients with psoriasis tend to have reduced high-density lipoprotein (HDL) concentrations [14]. Because the exact cause of psoriasis is unknown, several factors, including genetic, environmental, and immune-mediated inflammatory reactions, may be responsible [15,16]. A metabolic syndrome is a group of risk factors that includes central obesity, atherogenic dyslipidemia, glucose intolerance, and high blood pressure [17,18]. Individuals with psoriasis have increased chronic inflammation, which can exacerbate the severity of metabolic syndrome.

Lipid metabolism abnormalities and chronic inflammation are more common among patients with psoriasis and PsA. Hence, the primary objective of this study was to evaluate the serum concentrations of biochemical markers, such as lipid profile, fasting blood sugar (FBS), and IL-6, in each patient. Additionally, the study aimed to establish the relationship between these parameters and psoriasis severity, as measured by the PASI score.

## Materials and methods

The Department of Biochemistry at the Veer Surendra Sai Institute of Medical Sciences and Research, Burla, collaborated with the Departments of Dermatology and Community Medicine to conduct this institution-based cross-sectional study. The study lasted approximately eight months, from November 2022 to June 2023. The institutional ethics committee approved the study protocol under registration no. EC/NEW/INST/2021/2010. Before the study, written consent was obtained from participants. We followed the consecutive sampling method and enrolled 102 study subjects.

The Dermatology Department clinically diagnosed outpatients using ClASsification criteria for Psoriatic ARthritis. The severity of psoriasis lesions was assessed based on the psoriasis area severity index (PASI) score. Patients with a PASI score of less than 10 were classified as having mild psoriasis, while those with a PASI score greater than 10 were considered to have moderate-to-severe psoriasis [19].

Inclusion criteria

Clinically diagnosed cases of psoriasis, aged 18 to 50 years of either sex, who were willing to participate were included.

Exclusion criteria

Patients were excluded if they were under 18 years old, unwilling to give consent, or had acute illnesses such as fever, acute abdomen, or pregnancy. Additionally, those with a history of rheumatoid arthritis with rheumatoid factor positivity, malignancy, deep fungal or gonococcal infections, hepatorenal involvement, autoimmune diseases, cardiovascular diseases, or uncontrolled diabetes mellitus were excluded. The study excluded patients on systemic or oral hormonal therapy, retinoids, beta-blockers, or lipid-lowering drugs in the past six months.

Biochemical analysis

Following a 12-hour fast and adhering to all aseptic laboratory procedures, we extracted 5 mL of blood from the patient's antecubital veins. The sample was allowed to clot and then centrifuged at 3,000 revolutions per minute for 10 minutes. The serum was separated to evaluate the lipid profile and FBS using a fully automated analyzer (Cobas C311, Roche Diagnostics, Mannheim, Germany) and appropriate diagnostic kits per the manufacturer's instructions. Serum LDL-C was calculated using the Friedewald formula. The remaining serum sample was preserved at -70°C in a freezer for further use. Following the manufacturer's instructions, these frozen samples were used to estimate IL-6 levels using enzyme-linked immunosorbent assay (Erba, Transasia Bio-medicals Ltd., India).

Clinical and anthropometric parameters

We collected anthropometric data from the participants, including age, body weight, height, and body mass index (BMI). The severity score was graded on a scale from 0 to 4 for calculating the PASI score, where 0 = nil, 1 = mild, 2 = moderate, 3 = severe, and 4 = very severe. Each area was scored individually, and the four scores were combined into the final PASI. Additionally, the percentage of the area involved was scored as follows: 1 = ≤10%; 2 = 10%-29%; 3 = 30%-49%; 4 = 50%-69%; 5 = 70%-89%; 6 = >90% [19].

Statistical analysis

We analyzed the data using Statistical Package for the Social Sciences (SPSS) version 21.0 (IBM Corp., Armonk, NY), employing an unpaired t-test to assess the relationship between two variables. A p value of <0.05 was considered statistically significant. The correlation between specific lipid parameters, such as TC, HDL, and IL-6, with the PASI score was analyzed using Pearson’s correlation coefficient. Quantitative data were expressed as mean and standard deviation, while qualitative data were expressed in frequency and percentage.

## Results

Table 1 shows the clinical and demographic features of psoriasis patients. The present study included 102 samples, primarily of plaque psoriasis. Of these, 68 individuals (66.66%) had psoriasis alone, while 34 individuals (33.33%) had both psoriasis and arthritis. The mean ages of patients with psoriasis and PsA were 42.08 ± 5.59 and 43.10 ± 6.58 years, respectively. We found that parameters such as systolic blood pressure (p = 0.62), diastolic blood pressure (p = 0.29), and body surface area (BSA) (p = 0.38) were not significantly different in cases of psoriasis. However, in PsA, both the duration of the disease (11.35 ± 8.96, p < 0.05) and PASI scores (24.78 ± 3.44, p < 0.05) were significantly higher than those with psoriasis alone. Our study revealed a higher BMI in PsA patients, but this difference was not statistically significant (p = 0.15).

**Table 1:** Clinical and demographic parameters of study groups PsA: psoriatic arthritis; BMI: body mass index; SBP: systolic blood pressure; DBP: diastolic blood pressure; PASI: psoriasis area severity index; BSA: body surface area (% of body areas affected by psoriatic lesions)

Number of patients	Psoriasis alone (n = 68)	Psoriasis + PsA (n = 34)	p value
Sex (male/female)	46/22	23/11	0.98
Age (years)	42.08 ± 5.59	43.10 ± 6.58	0.90
BMI (kg/m^2^)	26.89 ± 4.38	28.69 ± 5.13	0.15
SBP (mm Hg)	129.22 ± 15.23	128.65 ± 16.95	0.62
DBP (mm Hg)	79.56 ± 13.50	84.86 ± 9.56	0.29
Psoriasis duration (years)	10.02 ± 8.35	11.35 ± 8.96	<0.05
PASI	9.68 ± 0.57	24.78 ± 3.44	<0.05
BSA	32.58 ± 13.25	39.65 ± 11.09	0.38

Table 2 displays the biochemical parameters. All atherogenic parameters, such as TC, TG, and low-density lipoprotein (LDL), were higher in PsA patients (p < 0.05) than those with psoriasis alone. The mean serum HDL concentration was significantly higher in psoriasis patients (43.68 ± 0.43, p < 0.05) than in PsA patients (37.2 ± 1.40). FBS and IL-6 concentrations were also higher in PsA patients, with mean values of 130 ± 4.24 mg/dL and 8.12 ± 1.02 pg/mL, respectively.

**Table 2:** Biochemical parameters among study participants PsA: psoriatic arthritis; SD: standard deviation; HDL: high-density lipoprotein; LDL: low-density lipoprotein; VLDL: very-low-density lipoprotein; FBS: fasting blood sugar; IL-6: interleukin 6

Parameter	Psoriasis alone (n = 68)	Psoriasis + PsA (n = 34)	p value
	Mean ± SD	Mean ± SD	
Total cholesterol (mg/dL)	187.26 ± 7.82	203.27 ± 9.36	<0.05
HDL (mg/dL)	43.68 ± 0.43	37.20 ± 1.40	<0.05
LDL (mg/dL)	112.70 ± 7.38	128.23 ± 9.12	<0.05
VLDL (mg/dL)	30.95 ± 4.17	36.93 ± 4.46	0.26
Triglycerides (mg/dL)	152.18 ± 14.34	185.89 ± 22.19	<0.05
FBS (mg/dL)	118.75 ± 3.75	130 ± 4.24	0.081
IL-6 (pg/mL)	5.94 ± 2.22	16.50 ± 5.27	<0.05

There were significant positive correlations between TC, TG, LDL, and IL-6 with the PASI score: r = 0.093 (p < 0.05), r = 0.080 (p < 0.05), r = 0.102 (p < 0.05), and r = 0.278 (p < 0.05), respectively. In PsA patients, HDL was negatively correlated with the PASI score (r = -0.036, p < 0.05). Psoriasis duration and BSA did not significantly correlate with the PASI score in either study group, as shown in Table 3.

**Table 3:** Association of serum IL-6, lipid profile, and clinical parameters with PASI score in study groups PsA: psoriatic arthritis; r: Pearson coefficient; HDL: high-density lipoprotein; LDL: low-density lipoprotein; VLDL: very-low-density lipoprotein; IL-6: interleukin 6; BSA: body surface area (% of body areas affected by psoriatic lesions); PASI: psoriasis area severity index ^a^Significant at <0.05

Parameters	Psoriasis alone (r value)	Psoriasis + PsA (r value)	p value
Total cholesterol (mg/dL)	0.057	0.093	<0.05
HDL cholesterol (mg/dL)	0.008	-0.036	<0.05
LDL cholesterol (mg/dL)	-0.013	0.102	<0.05
VLDL cholesterol (mg/dL)	0.128	0.057	<0.05
Triglycerides (mg/dL)	0.031	0.080	<0.05
IL-6 (pg/mL)	0.095	0.278	<0.05
Psoriasis duration	0.33	-0.09	0.865^a^
BSA	0.28	0.35	0.094^a^

## Discussion

Psoriasis is a significant dermatological disorder that affects large areas of the skin and the articular system. Chronic systemic inflammation is important because it increases the risk of cardiovascular disorders and metabolic syndrome. This inflammation is more severe in PsA due to additional joint involvement [20-22]. Patients with psoriasis have elevated levels of proinflammatory cytokines, such as IL-6 and tumor necrosis factor-alpha, contributing to systemic diseases [23].

The current study found that patients with psoriasis had elevated serum IL-6 levels; however, levels in PsA patients were significantly higher. Pietrzak et al. conducted a study that revealed elevated IL-6 levels in PsA compared with psoriasis alone [7], with values of 5.94 ± 2.22 and 16.50 ± 5.27, respectively (p < 0.05). The elevated serum IL-6 levels in patients with PsA were attributed to joint inflammation and increased PASI scores [7,24]. This study showed a significant correlation between IL-6 levels and PASI scores in patients with psoriasis, as indicated in Table 3 (0.095 < 0.05, 0.278, < 0.05). A previous study by Arican et al. found an increase in IL-6 levels among patients with psoriasis and a positive correlation with skin lesions (PASI) [9]. Laurent et al. concluded that the IL-6 receptor antagonist tocilizumab plays a role in treating psoriasis [25]. Similarly, Mease et al. found that clazakizumab is a safe and effective anti-IL-6 monoclonal antibody for treating people with active PsA [26].

This study evaluated the lipid profiles of patients with psoriasis and PsA. We found altered serum TC, TG, and LDL levels in both groups. However, psoriasis alone was associated with higher HDL levels. There was a significant positive correlation among TC, TG, and LDL with PASI in PsA. A previous study also found that people with PsA may have higher atherogenic parameters and lower HDL levels due to joint inflammation [18]. The low serum HDL level in PsA may trigger the formation of autoantibodies against HDL and apolipoprotein A1 (ApoA1), particularly in inflamed joints [24]. This could increase cardiovascular risk in psoriasis patients with arthritis [24]. Additionally, FBS values are higher in PsA than in psoriasis alone [25]. Thus, IL-6 plays a crucial role in the chronic inflammatory process and promotes atherogenic effects in psoriasis patients [27].

Limitations

The main limitation of the present study was its short duration as a cross-sectional study. The second limitation was the lack of a control group. To confirm our findings, larger scale, longer duration studies should be conducted.

## Conclusions

The present study concludes that there are variations in the lipid profile and increased IL-6 activity in patients with PsA compared to those with psoriasis. The study revealed that patients with higher atherogenic parameters (TC, TG, and LDL) also had increased IL-6 values. This suggests that IL-6 and dyslipidemia play roles in disease progression. Therefore, by measuring inflammatory markers (such as IL-6) and lipid profiles early in the disease process and implementing lifestyle modifications, such as maintaining a proper diet and engaging in regular exercise, we can employ preventive strategies for better management and help prevent complications in patients with psoriasis.

## References

[1] Griffiths CE, Barker JN (2007). Pathogenesis and clinical features of psoriasis. Lancet.

[2] Tucker LJ, Ye W, Coates LC (2018). Novel concepts in psoriatic arthritis management: can we treat to target?. Curr Rheumatol Rep.

[3] Kumar R, Sharma A, Dogra S (2014). Prevalence and clinical patterns of psoriatic arthritis in Indian patients with psoriasis. Indian J Dermatol Venereol Leprol.

[4] Kerschbaumer A, Fenzl KH, Erlacher L, Aletaha D (2016). An overview of psoriatic arthritis - epidemiology, clinical features, pathophysiology and novel treatment targets. Wien Klin Wochenschr.

[5] Pietrzak A, Michalak-Stoma A, Chodorowska G, Szepietowski JC (2010). Lipid disturbances in psoriasis: an update. Mediators Inflamm.

[6] Saggini A, Chimenti S, Chiricozzi A (2014). IL-6 as a druggable target in psoriasis: focus on pustular variants. J Immunol Res.

[7] Pietrzak A, Chabros P, Grywalska E (2020). Serum concentration of interleukin 6 is related to inflammation and dyslipidemia in patients with psoriasis. Postepy Dermatol Alergol.

[8] Balato A, Schiattarella M, Di Caprio R, Lembo S, Mattii M, Balato N, Ayala F (2014). Effects of adalimumab therapy in adult subjects with moderate-to-severe psoriasis on Th17 pathway. J Eur Acad Dermatol Venereol.

[9] Arican O, Aral M, Sasmaz S, Ciragil P (2005). Serum levels of TNF-α, IFN-γ, IL-6, IL-8, IL-12, IL-17, and IL-18 in patients with active psoriasis and correlation with disease severity. Mediators Inflamm.

[10] Grossman RM, Krueger J, Yourish D (1989). Interleukin 6 is expressed in high levels in psoriatic skin and stimulates proliferation of cultured human keratinocytes. Proc Natl Acad Sci U S A.

[11] Dowlatshahi EA, van der Voort EA, Arends LR, Nijsten T (2013). Markers of systemic inflammation in psoriasis: a systematic review and meta-analysis. Br J Dermatol.

[12] Baz K, Cimen MY, Kokturk A (2003). Oxidant / antioxidant status in patients with psoriasis. Yonsei Med J.

[13] Rocha-Pereira P, Santos-Silva A, Rebelo I, Figueiredo A, Quintanilha A, Teixeira F (2001). Dislipidemia and oxidative stress in mild and in severe psoriasis as a risk for cardiovascular disease. Clin Chim Acta.

[14] Azfar RS, Gelfand JM (2008). Psoriasis and metabolic disease: epidemiology and pathophysiology. Curr Opin Rheumatol.

[15] He L, Qin S, Dang L, Song G, Yao S, Yang N, Li Y (2014). Psoriasis decreases the anti-oxidation and anti-inflammation properties of high-density lipoprotein. Biochim Biophys Acta.

[16] Kawalec P, Malinowski KP, Pilc A (2016). Disease activity, quality of life and indirect costs of psoriatic arthritis in Poland. Rheumatol Int.

[17] Vanaclocha F, Belinchón I, Sánchez-Carazo JL (2014). Cardiovascular risk factors and cardiovascular diseases in patients with moderate to severe psoriasis under systemic treatment. PSO-RISK, descriptive study. Eur J Dermatol.

[18] Uczniak S, Gerlicz ZA, Kozłowska M, Kaszuba A (2016). Presence of selected metabolic syndrome components in patients with psoriasis vulgaris. Postepy Dermatol Alergol.

[19] Oh EH, Ro YS, Kim JE (2017). Epidemiology and cardiovascular comorbidities in patients with psoriasis: a Korean nationwide population-based cohort study. J Dermatol.

[20] Jones SM, Harris CP, Lloyd J, Stirling CA, Reckless JP, McHugh NJ (2000). Lipoproteins and their subfractions in psoriatic arthritis: identification of an atherogenic profile with active joint disease. Ann Rheum Dis.

[21] Li WQ, Han JL, Manson JE, Rimm EB, Rexrode KM, Curhan GC, Qureshi AA (2012). Psoriasis and risk of nonfatal cardiovascular disease in U.S. women: a cohort study. Br J Dermatol.

[22] Chandran V, Cook RJ, Edwin J (2010). Soluble biomarkers differentiate patients with psoriatic arthritis from those with psoriasis without arthritis. Rheumatology (Oxford).

[23] Nijsten T, Wakkee M (2009). Complexity of the association between psoriasis and comorbidities. J Invest Dermatol.

[24] Muramatsu S, Kubo R, Nishida E, Morita A (2017). Serum interleukin-6 levels in response to biologic treatment in patients with psoriasis. Mod Rheumatol.

[25] Laurent S, Le Parc JM, Clérici T, Bréban M, Mahé E (2010). Onset of psoriasis following treatment with tocilizumab. Br J Dermatol.

[26] Mease PJ, Gottlieb AB, Berman A, Drescher E, Xing J, Wong R, Banerjee S (2016). The efficacy and safety of clazakizumab, an anti-interleukin-6 monoclonal antibody, in a phase IIb study of adults with active psoriatic arthritis. Arthritis Rheumatol.

[27] Pal M, Febbraio MA, Whitham M (2014). From cytokine to myokine: the emerging role of interleukin-6 in metabolic regulation. Immunol Cell Biol.

